# The protease inhibitor JO146 demonstrates a critical role for CtHtrA for *Chlamydia trachomatis* reversion from penicillin persistence

**DOI:** 10.3389/fcimb.2013.00100

**Published:** 2013-12-18

**Authors:** Vanissa A. Ong, James W. Marsh, Amba Lawrence, John A. Allan, Peter Timms, Wilhelmina M. Huston

**Affiliations:** ^1^School of Biomedical Sciences, Institute of Health and Biomedical Innovation, Queensland University of TechnologyBrisbane, QLD, Australia; ^2^The Wesley Research Institute, Wesley HospitalAuchenflower, QLD, Australia; ^3^The Wesley Reproductive Medicine and Gynaecological Surgery Unit, The Wesley HospitalAuchenflower, QLD, Australia

**Keywords:** *Chlamydia*, persistence, HtrA/CtHtrA, protease inhibitor, protease

## Abstract

The *Chlamydia trachomatis* serine protease HtrA (CtHtrA) has recently been demonstrated to be essential during the replicative phase of the chlamydial developmental cycle. A chemical inhibition strategy (serine protease inhibitor JO146) was used to demonstrate this essential role and it was found that the chlamydial inclusions diminish in size and are lost from the cell after CtHtrA inhibition without formation of viable elementary bodies. The inhibitor (JO146) was used in this study to investigate the role of CtHtrA for penicillin persistence and heat stress conditions for *Chlamydia trachomatis*. JO146 addition during penicillin persistence resulted in only minor reductions (~1 log) in the final viable infectious yield after persistent *Chlamydia* were reverted from persistence. However, JO146 treatment during the reversion and recovery from penicillin persistence was completely lethal for *Chlamydia trachomatis*. JO146 was completely lethal when added either during heat stress conditions, or during the recovery from heat stress conditions. These data together indicate that CtHtrA has essential roles during some stress environments (heat shock), recovery from stress environments (heat shock and penicillin persistence), as well as the previously characterized essential role during the replicative phase of the chlamydial developmental cycle. Thus, CtHtrA is an essential protease with both replicative phase and stress condition functions for *Chlamydia trachomatis*.

## Introduction

*Chlamydia trachomatis* is a unique obligate intracellular bacterial pathogen. The organism is typified by a bi-phasic developmental cycle. This cycle consists of an infectious extracellular form, termed the elementary body (EB), and an intracellular replicative form termed the reticulate body (RB), which divides by binary fission prior to converting back to the infectious progeny (reviewed, Adbelrahman and Belland, 2005). The intracellular form is found inside a unique vacuole inside the host cell that is called the inclusion vacuole. In addition to these two forms, the organism has a “latent” like phase of intracellular growth. This is termed persistence that is defined as viable but non-cultivatable *Chlamydia.* Persistent *Chlamydia* (also called aberrant bodies), are morphologically distinct from the active replicating form with only a few cells visible per inclusion which are much larger size) (Moulder, 1993; Byrne et al., 2001; Hogan et al., 2004; Wyrick, 2010). This ability to become persistent is thought to provide the organism with a survival mechanism to avoid any conditions where they would be unable to survive. Hence, persistence is induced by immune pressure, amino acid deprivation, penicillin, iron limitation, or the presence of other intracellular pathogens (Beatty et al., 1993, 1994a,b; Coles et al., 1993; Byrne et al., 2001; Wyrick and Knight, 2004; CDC, 2005–2009/2010; Deka et al., 2006). Whilst there are numerous means of inducing persistence and the chlamydial cellular morphology appears similar for each of these, it is clear that there are distinct transcriptional and protein profiles associated with the different forms of persistence (reviewed, Wyrick, 2010).

Amino acid deprivation has been shown to induce persistence, which was able to be restored by cysteine and isoleucine (Coles et al., 1993; Harper et al., 2000). The best characterized mechanism of persistence is that induced by IFN-γ (interferon-gamma). IFN-γ (secreted by immune cells) induces a large range of responses in the epithelial cell (Beatty et al., 1993, 1994a). One of the proteins that is highly induced in human epithelial cells in response to IFN-γ is IDO1 (indoleamine 2,3-dioxygenase) (Beatty et al., 1993, 1994a; Ibana et al., 2011). This enzyme catabolizes the host cell tryptophan resulting in reduced tryptophan supply for the auxotrophic pathogen. *C. trachomatis* persistence induced by IFN-γ is able to be reverted by the removal of IFN-γ and addition of tryptophan. IFN-γ aberrant bodies are typified by a loss of expression of genes required for cytokinesis with continuing chromosomal replication (Byrne et al., 2001; Belland et al., 2003). Another commonly used laboratory model of persistence is that which occurs in response to cell wall targeting antibiotics. Models of this form of persistence typically involve penicillin. Penicillin persistence has been described to result in the *Chlamydia* cells rapidly ceasing cellular division, whilst chromosomal and plasmid replication continue at the same rate regardless of the presence or absence of penicillin (Byrne et al., 2001). The removal of the penicillin then allows reversion, which occurs via an RB budding from the aberrant body, with this only productively occurring in some inclusions (Skilton et al., 2009).

Recently, our team identified a serine protease inhibitor (JO146) against *C. trachomatis* HtrA (CtHtrA) which was lethal when added to cultures during the mid-replicative phase (Gloeckl et al., 2013). The compound is a serine protease inhibitor, which is a tri-peptide with a war-head motif. The compound was firstly identified to be specific to CtHtrA using *in vitro* CtHtrA protease assays (Gloeckl et al., 2013). JO146 was demonstrated to be lethal when added to cultures during the replicative phase of development but not when added early or late during the developmental cycle (Gloeckl et al., 2013). HtrA in many bacteria is a periplasmic protease involved in cell surface protein assembly and extracytoplasmic protein maintenance (reviewed, Clausen et al., 2011). This function is also supported by our data to date for *Chlamydia* HtrA (Huston et al., 2008), and it is likely that this extracytoplasmic protein protection role is particularly critical during the chlamydial penicillin persistence model. Previously, we and others have reported that CtHtrA is highly expressed during penicillin persistence lab models and down regulated during IFN-γ persistence (Belland et al., 2003; Mukhopadhyay et al., 2006; Huston et al., 2008). Therefore, in this project we aimed to test the hypothesis that CtHtrA is essential during penicillin persistence using the CtHtrA inhibitor JO146.

## Materials and methods

### *Chlamydia* culture

**Figure 1:**
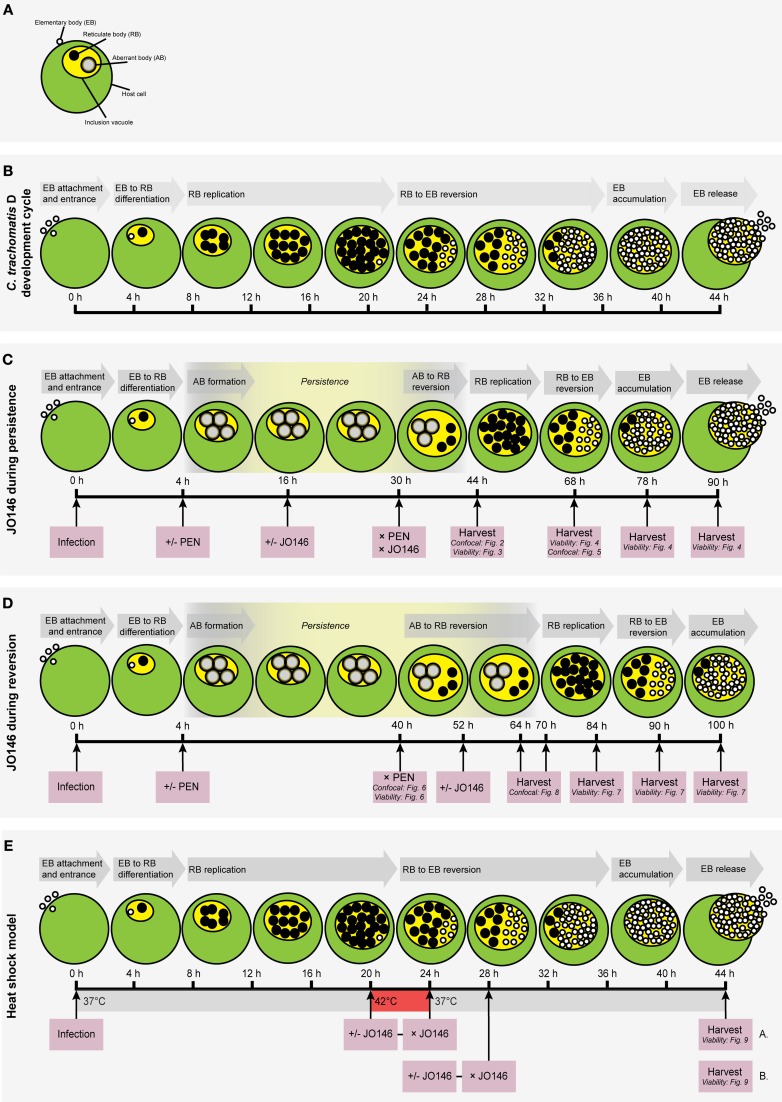
**Flow chart diagrams of methodologies used in this manuscript with cartoon representations of the expected growth phases of the *Chlamydia*. (A)** Key to the components of a *Chlamydia-*infected host cell. Small, open circles: elementary bodies (EB); large black circles: reticulate bodies (RB); large gray circles: aberrant bodies (AB); green circle: host cell; yellow circle: chlamydial inclusion vacuole. **(B)** The *Chlamydia trachomatis* (serovar D) development cycle is represented by the cartoon. The relative time-points are shown below the cells and each stage of the cycle is shown in gray arrows above the cells. **(C)** Experimental conditions used to assess the impact of JO146 addition during penicillin persistence. **(D)** Experimental plan to determine the impact of JO146 on *C. trachomatis* reversion from persistence. **(E)** Experimental plan to use the JO146 inhibitor to determine the role of CtHtrA for viability of *C. trachomatis* during heat stress conditions. The pink boxes represent the experimental actions taken, with arrows extending to the specific time-point for each action. The viability and/or morphological experiments associated with specific time-points are included in small type in the relevant pink boxes. The gray arrows indicate the stage of the development cycle represented by the cartoon. “PEN” represents penicillin. “+/−” indicates that separate experiments were conducted with or without the addition of penicillin (“+/− PEN”) or the JO146 inhibitor (“+/− JO146”). “× PEN” indicates that penicillin was removed in the experiments where it was added. The yellow highlighted section of the developmental cycle indicates the expected presence and relative duration of persistence when induced by the addition of penicillin at 4 h PI in the persistence experiments. The cartoon representations of the expected chlamydial developmental cycle phases and the associated time points under these experimental conditions are based on previously published data (Byrne et al., 2001; Miyairi et al., 2006; Skilton et al., 2009).

*Chlamydia trachomatis* (serovar D UW-E/Cx) was routinely cultured in HEp-2 cells in DMEM (Dulbecco's modified eagle medium), 5% FCS (fetal calf serum), 37°C, 5% CO_2_. All cultures were conducted at a 0.3 multiplicity of infection (MOI). A summary of the experimental methods used in this study is shown in Figure 1. Penicillin persistence was established by the addition of 100 U/ml of penicillin at 4 h PI (hours post-infection) and JO146 was added at 16 h PI to determine the impact of JO146 treatment during persistence. In order to measure the impact of JO146 on the ultimate viability, the cultures were allowed to revert from persistence by the removal of penicillin. Penicillin was removed by three sequential rounds of media washes and medium was replaced with penicillin-free media at 30 h PI. Viable infectious yield was measured at 68, 78, and 90 h PI. Cultures were also monitored for viability at 44 h PI without the removal of the penicillin to demonstrate lack of viability consistent with persistence (in conjunction with the ability to subsequently rescue these same culture conditions to detectable viability by penicillin removal). Control cultures with no JO146 were included for each experiment and with the solvent DMSO (dimethyl sulfoxide). In order to assess the impact of JO146 on *Chlamydia* during reversion from penicillin persistence, a separate experiment was conducted where persistence was induced in the cultures using penicillin (4 h PI, 100 U ml-1); at 40 h PI the penicillin was removed (washes and media change). At 52 h PI when reversion is likely to be underway in most inclusions, JO146 was added to the cultures (concentrations as indicated on the figures). Reversion is very asynchronous from this form of persistence. Reversion has been reported to take 10–20 h for *C. trachomatis* L2 which has similar (slightly faster) growth kinetics to the strain used in this study. Therefore, 52 h PI is the most logical choice to target reversion based on available data (Skilton et al., 2009). The cultures were harvested and viability was determined at 84, 90, and 100 h PI. Control cultures with no JO146 treatment were included for each experiment. Cultures for immunocytochemistry were conducted on glass coverslips, using the method previously described (Huston et al., 2008).

A heat shock model was used in conjunction with JO146 treatment to evaluate the role of CtHtrA for chlamydial viability during heat stress. Heat shock was conducted at 20 h PI for 4 h (20–24 h PI), as we have previously shown an increase in CtHtrA protein at this time point during heat shock (Huston et al., 2008). JO146 was added at 20 h PI, immediately prior to heat shock at 42°C, 5% CO_2_ for 4 h. At the conclusion of the 4 h heat shock, JO146 was removed from the cultures by three washes in 37°C pre-warmed media, prior to returning the culture to 37°C for the remainder of the developmental cycle. In a separate experiment, the role of CtHtrA during recovery from heat shock was also analyzed by the addition of JO146 immediately (at 24 h PI) after the 4 h heat shock treatment and removal of the compound by media washes (three) at 28 h PI. The cultures were harvested at 44 h PI and viable infectious yield was determined (see Figure 1 summary).

### Determination of chlamydial viability

*Chlamydia* viable infectious yield was determined by serial dilution of cultures and infection on HEp-2. At 30 h PI the monolayers were fixed and immunocytochemistry was conducted to enable counting of inclusions. The final results are presented as inclusion forming units per ml (IFU ml^−1^). Statistical analysis was performed using PRISM (GraphPad Software Inc). Results are expressed as mean, with error bars representing standard error of the mean. Two-Way analysis of variance (ANOVA) with a *post-hoc* Bonferroni multiple comparison tests was used to assay the statistical differences relative to the DMSO control.

### Immmuncytochemistry and microscopy

Cultures were fixed using 4% paraformaldehyde-PBS (phosphate buffered saline) for 15 min, and then blocked in 1% BSA-PBS overnight at 4°C. The primary antibody (*C. trachomatis* MOMP (major outer membrane proteim), Biodesign International) was added at 1:500 dilutions in 1% BSA (bovine serum albumin) in PBS with 2μL/coverslip phalloidin 594 (Invitrogen) and 1:40,000 dilution of DAPI (diaminidino phenylindole). Coverslips were incubated for 2 h at room temperature. Three washes with 0.2% Tween 20 in PBS were conducted prior to addition of the secondary antibody (goat anti-mouse IgG H+L-Alexa Flour 488, Invitrogen) at 1:2000 dilutions in 1% BSA in PBS. Coverslips were then washed 4 times with 0.2% Tween 20-PBS and were suspended in PBS prior to mounting with ProLong Gold (Invitrogen). Cultures fixed for immunocytochemistry were examined using the Leica SP5 confocal microscope. Images were prepared using the supplied Leica software suite. Sizes of inclusions at 44 h PI were measured to determine the effect of the inhibitor compound during penicillin-induced persistence using the Leica application suite.

## Results

### JO146 addition to *C. trachomatis* HEp-2 cultures during penicillin persistence results in a reduced viable yield

Due to the bi-phasic nature of the chlamydial developmental cycle it is not possible to measure the immediate impact on viability during the replicative phase of growth. Therefore, for each of these experiments we have assessed the viability once elementary bodies are formed, either at the conclusion of the developmental cycle or once reversion from persistence and development of elementary bodies has occurred. This also means that we can confirm that the cultures are persistent by detection of loss of viability in the persistent cultures when control cultures are demonstrated to have viable elementary bodies (as long as viability was subsequently restored by removal of the persistence inducing agent). We first wanted to monitor the impact of JO146 addition during persistence when aberrant bodies are present, hence for this experiment we added JO146 during persistence at 16 h PI (penicillin was added at 4 h PI to induce persistence) and one set of cultures were harvested to measure viability and also fixed and examined by confocal microscopy at 44 h PI. A second set of cultures were media changed at 30 h PI to remove both the JO146 and penicillin and harvested at 68, 78, and 90 h PI (or 38, 48, 60 h after penicillin removal) to allow time for reversion from persistence and elementary body formation (as outlined in Figure 1). These cultures were tested for viability and morphology was examined using confocal laser scanning microscopy.

**Figure 2:**
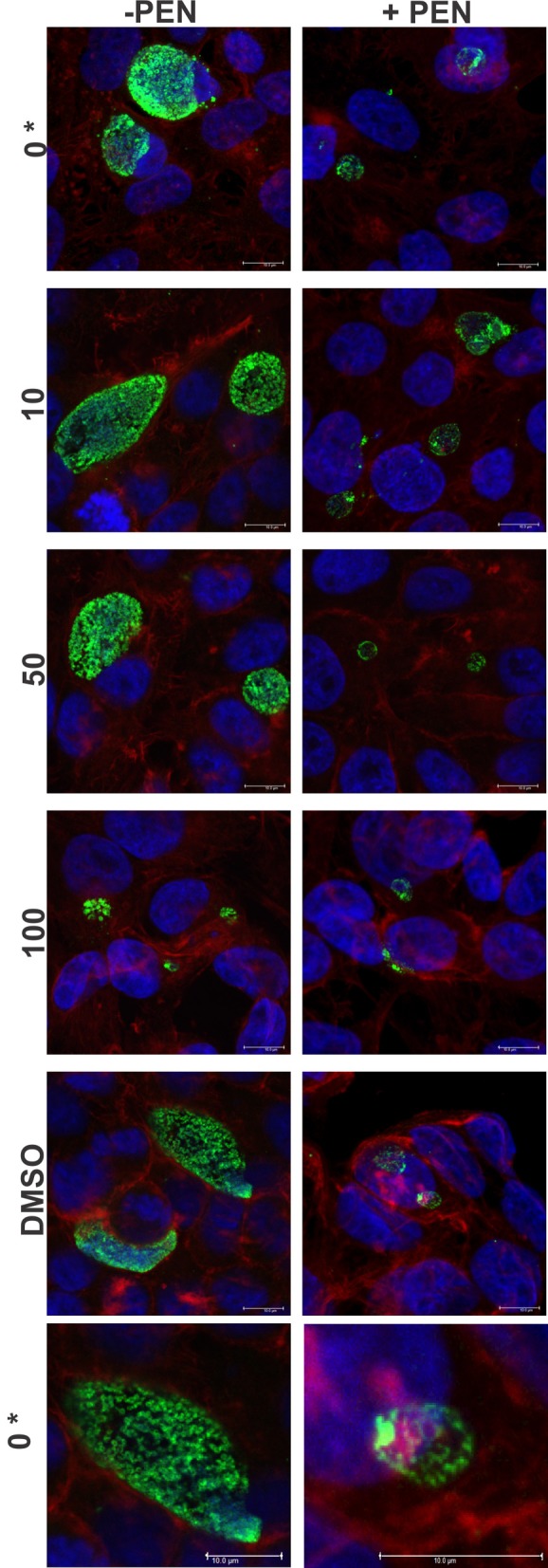
**Confocal microscopy images of JO146 treated cultures during penicillin persistence at 44 h PI (+PEN: penicillin added, −PEN: no penicillin).** The figure shows representative images from confocal laser scanning microscopy of cultures fixed at 44 h PI, 100 U ml^−1^penicillin was added at 4 h PI. Representative images from the control culture shown in the left column (−PEN). Penicillin treated conditions are shown in the right column. JO146 treatment conditions are indicated to the right (0, 10, 50, 100μM, DMSO). The final images at the bottom are enlarged images of the controls which have both had equal contrast adjustments to make the differences more apparent (0μM JO146 enlarged). The image colors are as follows, green: MOMP (major outer membrane protein) is green, blue: cell nucleus (DAPI), and red: β-actin. Scale bar (bottom right) indicates 10 mm. ^*^Indicates zoomed in images from the 0μM conditions are shown to allow closer examination of morphology.

**Figure 3:**
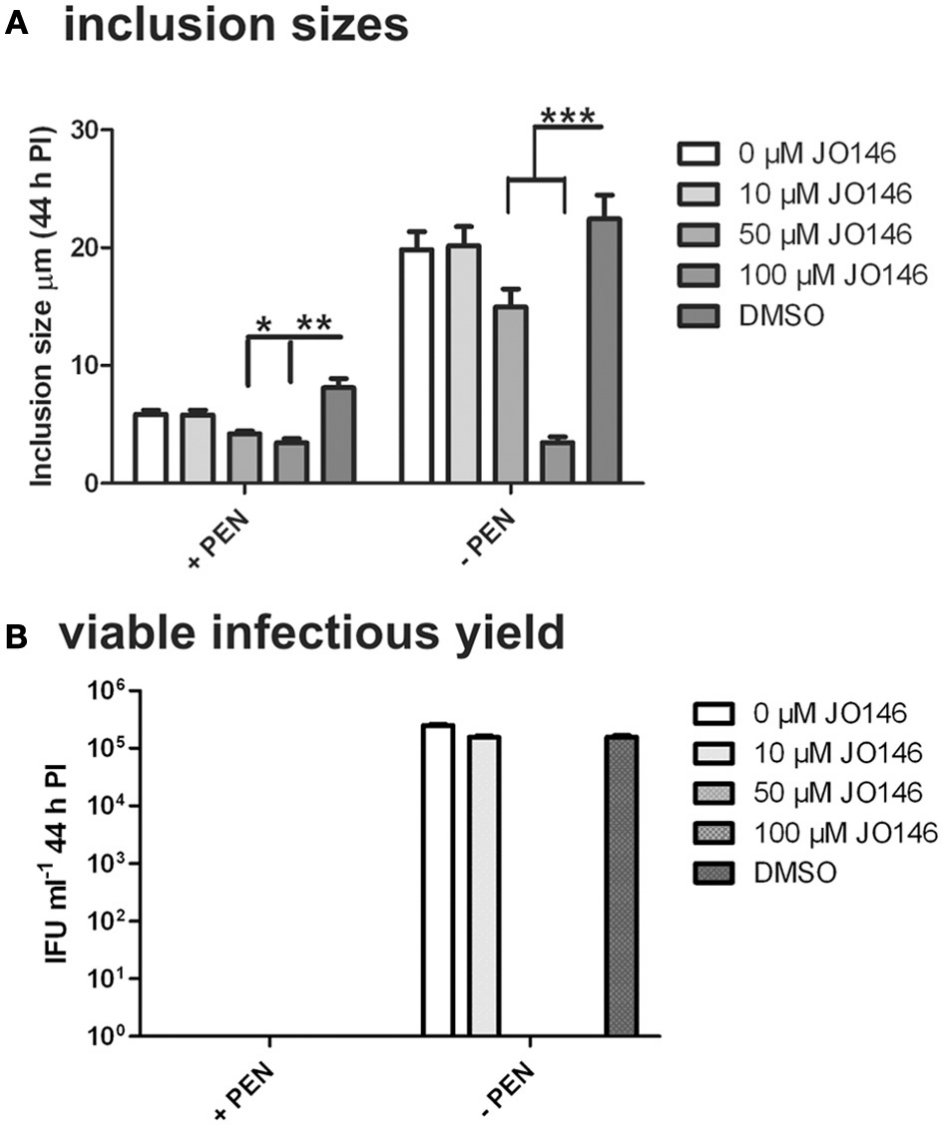
**Inclusion sizes and viable infectious yield during penicillin persistence at 44 h PI with and without JO146 treatment.** The figure shows morphological analysis of cultures during penicillin persistence and controls by measuring inclusion sizes. The viable infectious yield (44 h PI) in the presence or absence of 100 U ml^−1^ penicillin (4 h PI) is shown on the graph. **(A)** Inclusion sizes are shown from each condition inclusion sizes were measured from independent coverslips, *n* = 20. **(B)** Viable infectious yield with and without penicillin are shown graphically (*n* = 27). Statistics were conducted using Two-Way ANOVA relative to DMSO controls, *p* > 0.5^*^, *p* > 0.01^**^, *p* > 0.001^**^, *P* > 0.0001^***^.

The cultures were firstly confirmed to be persistent at 44 h PI by monitoring viability and impact of JO146 treatment in the presence and absence of penicillin. As shown in Figure 2, the cultures treated with penicillin had much smaller inclusions compared to the controls at 44 h PI. The inclusions were also much less populated with cell shaped bodies consistent with a persistent phenotype (Figure 2 right column penicillin compared to control left column). The increasing concentrations of JO146 resulted in a decreased inclusion vacuole size for both the penicillin treated and control cultures (Figure 3A). The penicillin treated cultures were confirmed to be persistent by a lack of viable EBs at 44 h PI (Figure 3B) and supported by restoration of viability in subsequent experiments.

**Figure 4:**
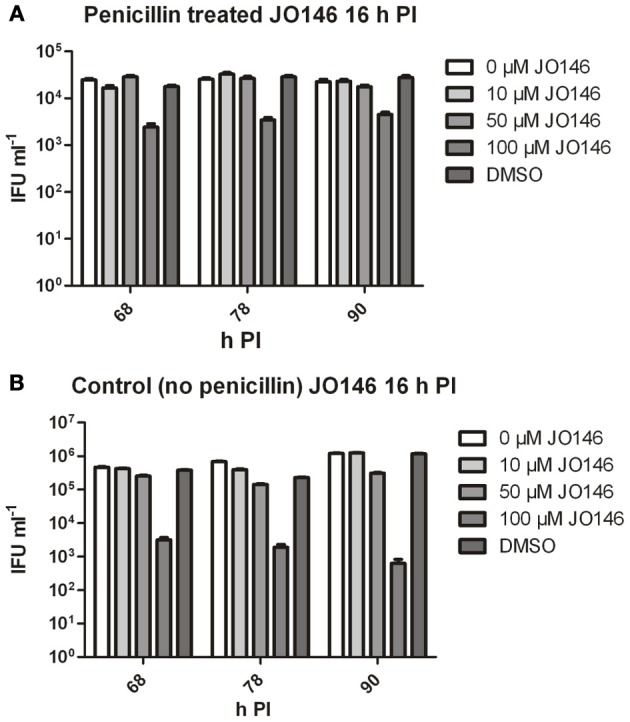
**Viable infectious yield of *C. trachomatis* after treatment with JO146 during penicillin persistence.** The figure shows viable IFU ml^−1^ from each culture condition at several time points after reversion was commenced (penicillin removal at 30 h PI). Cultures were treated with penicillin 100 U ml^−1^ at 4 h PI, and with JO146 at 16 h PI (concentrations indicated by the gray shading, see key to the right of each graph). Penicillin was removed from the cultures at 30 h PI. **(A)** Viable infectious yield from penicillin treated and restored cultures. **(B)** Viable infectious yield from control cultures which did not have penicillin added. Statistics were conducted using Two-Way ANOVA relative to DMSO controls.

The viable infectious yield during the reversion from persistence from the cultures with and without JO146 treatment was determined [during reversion at 68, 78, and 90 h PI, when we expect to see recovery to viable elementary bodies (Skilton et al., 2009)]. As expected, and consistent with our previously published data, JO146 was completely lethal on the control (non-persistent) culture at 50 and 100μM, when viability was measured at 44 h PI (Figure 3B) with some recovery of viability observed at the later time points (extended culture conditions, Figure 4B), as consistent with our previous work (Gloeckl et al., 2013). In contrast, JO146 was not lethal when it was added at the same time point during penicillin induced persistence (Figure 4A). Cultures were treated with JO146 during persistence then subsequently rescued by media change (30 h PI) to allow the formation of EBs (measured as viable infectious yield). JO146 treatment during the persistence phase resulted in a relatively minor loss of detectable viability, with approximately a 1 log reduction of viable yield observed at 100μM JO146 when EBs were able to be detected at 68, 78, and 90 h PI (Figure 4A). The control cultures which were not persistent showed ~2–3 log reductions in viability with 100μM JO146 treatment at the extended culture times of 68–90 h PI (Figure 4B). This observation of reduced effectiveness of JO146 over extended culture conditions is consistent with our previous data (Gloeckl et al., 2013).

**Figure 5:**
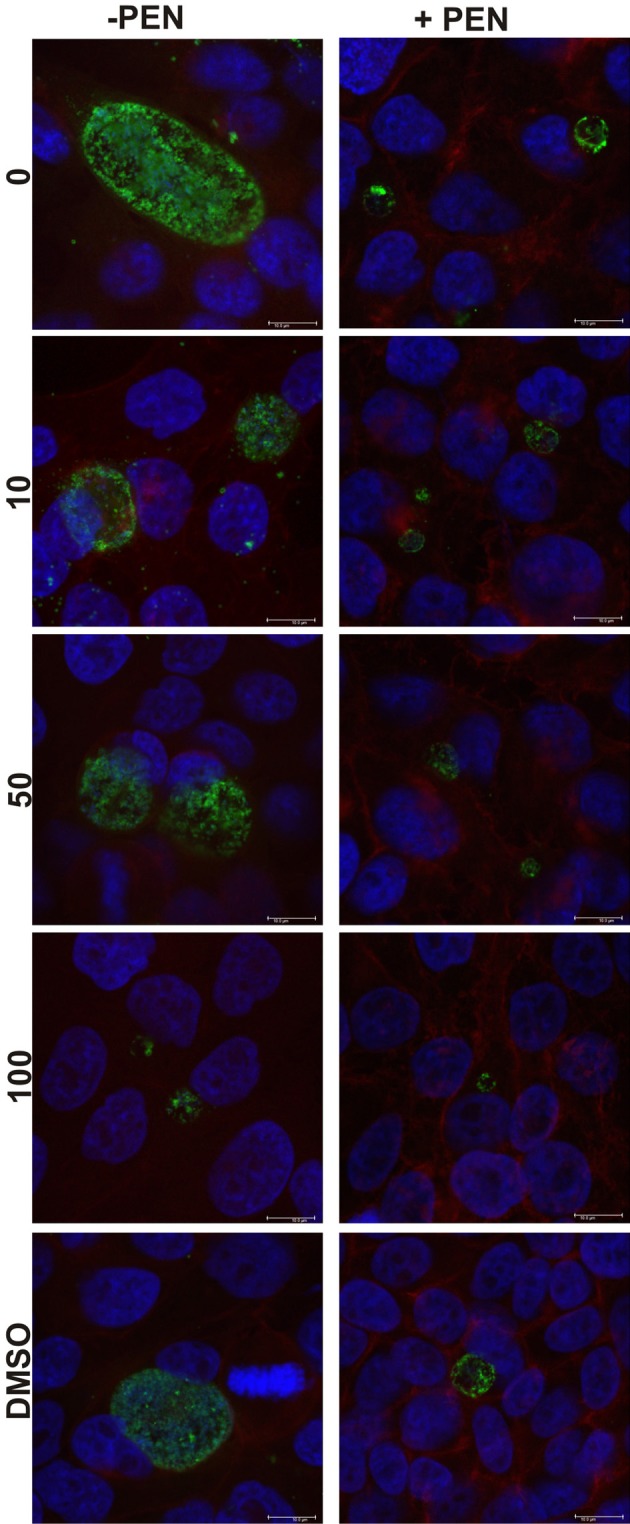
**Confocal microscopy images of *C. trachomatis* cultures at 68 h PI, or 38 h after penicillin reversion commenced.** Cultures were treated with penicillin 100 U ml^−1^ (right column) at 4 h PI, and with JO146 at 16 h PI (concentrations indicated by the gray shading, legend to right of each graph). Penicillin was removed from the cultures at 30 h PI. The image colors are as follows, green: MOMP (major outer membrane protein) is green, blue: cell nucleus (DAPI), and red indicates β-actin. Scale bar (bottom right) indicates 10μm.

The cultures were monitored by immunofluorescence during the reversion period and representative images from 68 h PI are shown in Figure 5 (left column control, right column penicillin). The penicillin treated cultures had smaller inclusions with different appearance (likely indicating there are still aberrant bodies present) at 68 h PI, with the inclusions generally appearing smaller in the presence of 100μM JO146 (Figure 5, right column). The control cultures (no penicillin treatment) also showed a JO146 concentration dependent reduction of the inclusion sizes (Figure 5, left column). However, even though the inclusions appeared markedly smaller when recovering from penicillin persistence at 68 h PI, there was only ~one log reduction in viable EB yield compared to the controls (Figure 4A).

### JO146 addition to *C. trachomatis* HEp-2 cultures during reversion from penicillin persistence is lethal

The mechanism of reversion from penicillin persistence has been described to be very asynchronous; with gradual budding of “normal” RBs from the aberrant persistent forms in the inclusion over 10–20 h after penicillin was removed (Skilton et al., 2009). These budded RBs are then thought to undergo replication by binary fission prior to conversion to the infectious elementary body form. Given the highly asynchronous nature of this reversion, at any one time there will be cells undergoing reversion and other cells undergoing replication at the same time, meaning that it is not possible to uncouple reversion from replication. We established penicillin persistent cultures and then commenced reversion by washing and media change at 40 h PI. 12 h (52 h PI) after reversion was commenced we added JO146 and monitored the formation of viable infectious elementary bodies at 84, 90, and 100 h PI.

**Figure 6:**
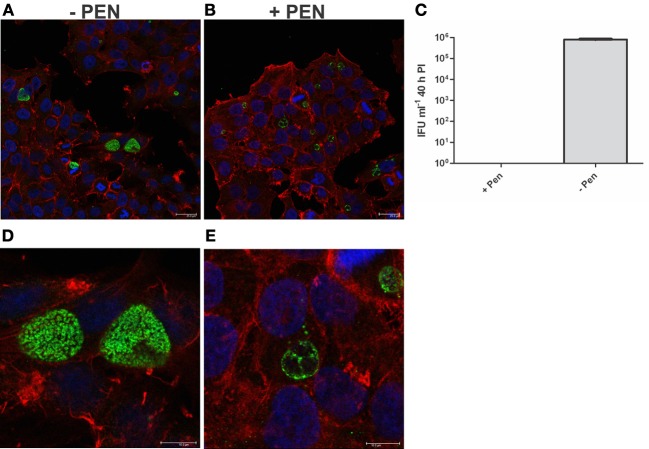
**Penicillin persistence cultures prior to commencement of reversion are not viable and are morphologically consistent with persistence.** Cultures were treated with penicillin 100 U ml^−1^ at 4 h PI. **(A)** Confocal microscopy image of cultures at 40 h PI in the presence of penicillin (+PEN) **(B)** Confocal microscopy image of cultures at 40 h PI in the absence of penicillin (−PEN). The scale bar indicates 25μm. **(C)** Viable infectious yield of the corresponding cultures (*n* = 27). **(D)** Enlarged area of **A**: confocal microscopy image of cultures at 40 h PI in the presence of penicillin (+PEN). **(E)** Enlarged area of **B**: confocal microscopy image of cultures at 40 h PI in the absence of penicillin (−PEN). The contrast has been equally adjusted on **D,E** to improve the visibility of the morphologies present.

**Figure 7:**
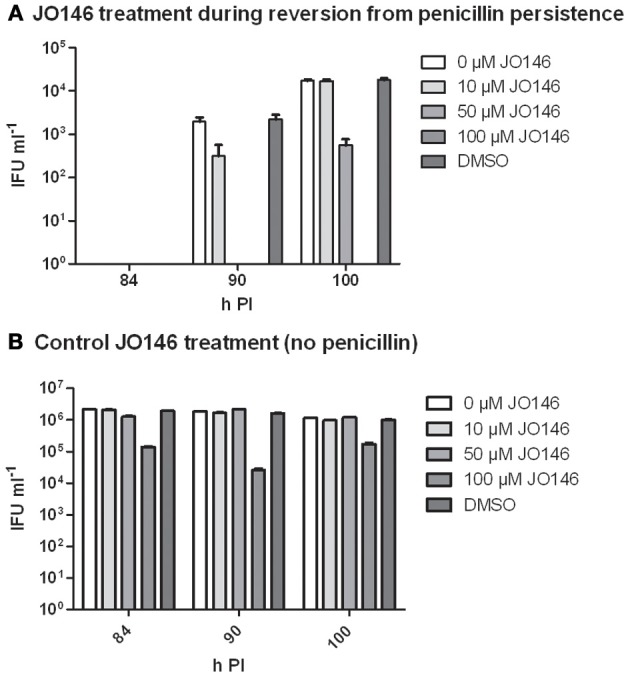
**Viable infectious yield when JO146 was added during the reversion from penicillin persistence.** Cultures were treated with penicillin 100 U ml^−1^ at 4 h PI. Penicillin was removed at 40 h PI and JO146 treatment was commenced at 52 h PI. **(A)** JO146 was added 12 h after penicillin persistence reversion was commenced (i.e., at 52 h PI). **(B)** Control cultures that were not persistent with JO146 treatment also conducted at 52 h PI. The graphs show the viable infectious yield at several time points after reversion from persistence was commenced (*n* = 27). JO146 concentration is indicated by the gray shading (legend to the right of each graph).

Firstly, we confirmed that the cultures were persistent at 40 h PI by measuring viability and examining the morphology of the cultures using confocal laser scanning microscopy. As shown in Figure 6, the penicillin treated cultures were not viable at 40 h PI (Figure 6C) and morphologically the inclusions were smaller and appeared to have large cellular forms present inside each inclusion (Figures 6A,B). The penicillin persistent cultures were washed to remove penicillin to commence reversion at 40 h PI. JO146 was added to these cultures 12 h after commencement of reversion (i.e., 52 h PI). Viable infectious yield was then measured over time from the cultures. As shown in Figure 7A no viable *Chlamydia* were detected at 84 h PI from the persistence reversion cultures (44 h after penicillin was removed), however, at 90 and 100 h PI viable EBs were detected. The JO146 treatment of 100μM JO146 was lethal to the cultures undergoing reversion (Figure 7A). In contrast, the cultures which were not penicillin persistent showed only minor reductions in viability due to the addition of JO146 (Figure 7B). These control cultures were likely either mainly in elementary body form or in the early stages of infection when JO146 was added (early or very late developmental cycle, or a mix of both), based on the morphological appearance of the inclusions and what we know about the timing of the chlamydial developmental cycle. We previously demonstrated that JO146 was less effective for both of these developmental phases (Gloeckl et al., 2013), so these results are consistent with what might be expected. Therefore, these data indicate that during reversion from penicillin persistence and recovery of viability, JO146 treatment is completely lethal for *Chlamydia*.

**Figure 8:**
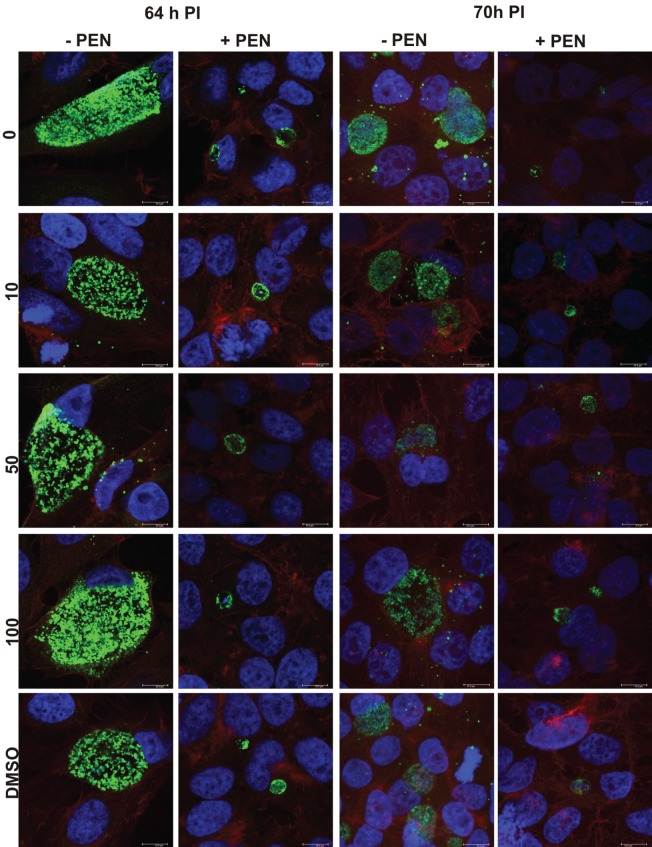
**Confocal microscopy images of penicillin persistent cultures and controls during reversion from persistence at 64 and 70 hPI.** Cultures were treated with penicillin 100 U ml^−1^ at 4 h PI. Penicillin was removed at 40 h PI and JO146 treatment was commenced at 52 h PI. Controls with no penicillin first and third column, penicillin conditions second and fourth columns. JO146 concentrations are indicated to the right. The image colors are as follows, green: MOMP (major outer membrane protein) is green, blue: cell nucleus (DAPI), and red: β-actin. Scale bar (bottom right) indicates 10μm.

We monitored the appearance of the cultures using immunocytochemistry and confocal laser scanning microscopy during the reversion from persistence to monitor the impact of JO146 on inclusion morphology at 64 and 70 h PI (12 and 18 h after JO146 addition). In this case we observed no obvious decrease in the inclusion size relating to JO146 treatment (Figure 8, second and fourth column), and as expected the inclusions from the persistent cultures were much smaller than those in the controls (Figure 8 controls first and third column).

### JO146 treatment is lethal during heat stress and recovery from heat stress for *Chlamydia*

**Figure 9:**
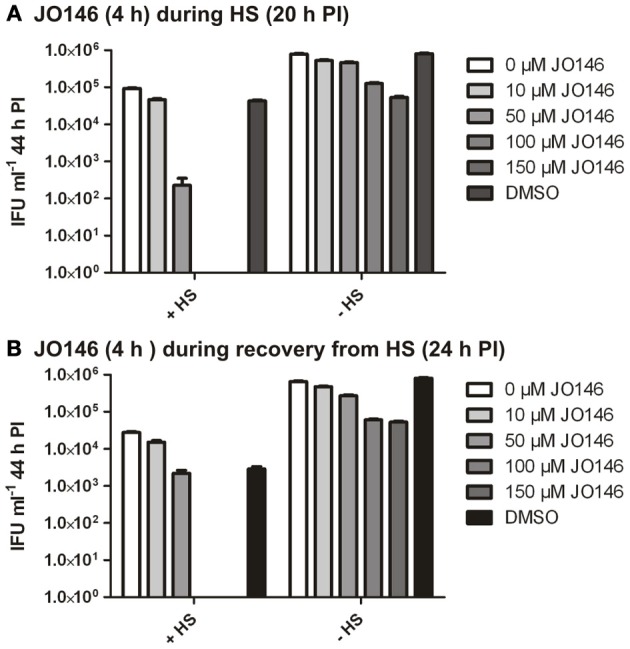
***C. trachomatis* viable infectious yield at 44 h PI after 4 h heat shock with JO146 treatment (20–24 h PI) or JO146 treatment during recovery from heat shock (24–28 h PI). (A)** The viable infectious yield after JO146 treatment for 4 h at 20 h PI with and without heat shock at 42°C. **(B)** Viable infectious yield after JO146 treatment for 4 h during recovery from heat shock (24–28 h PI) (*n* = 27). The concentration of JO146 is indicated by the gray scale bars, legend to the right of each graph.

In many bacteria, HtrA is widely acknowledged to be a stress response protease as well as being involved in crucial functions in outer membrane protein assembly and general protein maintenance (reviewed, Clausen et al., 2011). Accordingly, we used JO146 to evaluate the role of CtHtrA during heat stress and also during recovery from heat stress. We had previously shown increased protein levels of CtHtrA during a heat stress model, at 20 h PI, and this is also the phase of the developmental cycle at which we already know JO146 is effective (Huston et al., 2008; Gloeckl et al., 2012). *C. trachomatis* cultures (20 h PI) were heat stressed for 4 h in a 42°C 5% CO_2_ incubator prior to subsequent restoration to 37°C and completion of the developmental cycle. JO146 was added either during the heat shock and subsequently removed, or during the 4 h of post heat shock recovery and subsequently removed. The impact of JO146 treatment and the heat shock conditions were evaluated by determining the viable infectious yield at 44 h PI. In our previous work using JO146 we demonstrated that the compound needed to be present for longer than 4 h for lethality, therefore, during this experiment we used a higher concentration of 150μM. As shown in Figure 9A, the presence of JO146 during heat shock was completely lethal at 100 and 150μM. 50μM JO146 treatment during the 4 h heat shock also resulted in a marked loss of chlamydial viability (>2 log) (Figure 9A). Some JO146-induced reduction in viability was also observed in the controls which were not heat shocked (Figure 9A, indicated on *x* axis), and this is consistent with our previous observations that JO146 needs to be present throughout the replicative phase (not for 4 h only) to be completely lethal (Gloeckl et al., 2013).

JO146 treatment during the first 4 h of recovery from heat shock was also completely lethal at higher concentrations (100μM and 150μM), with a minor impact on viable yield observed with 50μM JO146 (Figure 9B). There was a more noticeable reduction in viability for the heat shocked cultures compared to the controls during this experiment (Figure 9B, conditions indicated on *x* axis). However, in spite of these differences it is clear that JO146 treatment both during heat stress and recovery from was lethal for *Chlamydia*, with a more marked impact at lower concentrations during the heat stress.

## Discussion

HtrA is an essential maintenance protease and chaperone for many bacteria. The protein conducts diverse roles in bacterial pathogenesis from being essential for outer membrane protein assembly (Purdy et al., 2007), to stress response and survival (Lipinska et al., 1989), cleavage of host proteins (Hoy et al., 2010) and intracellular infection survival (Pedersen et al., 2001). We previously used a chemical inhibition strategy to establish that *Chlamydia* HtrA (CtHtrA) is essential during the replicative phase of intracellular development. Using JO146 we found that CtHtrA inhibition at 16 h PI resulted in the reduction of inclusion sizes and eventually the inclusions were lost from the host cells with no detectable production of viable elementary bodies (Gloeckl et al., 2013). This data supports an essential role for CtHtrA during the replicative phase of chlamydial development. However, given the multi-tasking nature of HtrA already described for many other bacteria (reviewed, Clausen et al., 2011), we set out to test the hypothesis that CtHtrA is essential for heat stress conditions and during persistence models.

The data we present demonstrates that CtHtrA is essential for the reversion and recovery to viability for penicillin persistence and is also essential during heat stress and recovery from heat stress. The heat stress model is clearly likely to involve extra-cytoplasmic protein stress which will require both the protease activity and chaperone activity of CtHtrA. Clearly CtHtrA is essential, either for stress response or restoration of protein biogenesis, during the reversion and recovery to EBs from penicillin persistence. The recovery from penicillin persistence is very asynchronous and so it was not possible to uncouple restoration from persistence and the subsequent replication of the restored RBs. Therefore, it is possible that the impact of JO146 in this experiment was on the replication of recovered RBs or the recovery from penicillin persistence to reticulate bodies, or both. However, 44 h after reversion was commenced no viable EBs were detectable but they were at 50 h, which does suggest that the JO146 treatment was during the time frame likely to correspond with the majority of the population still undergoing reversion from persistence.

Based on the lack of lethality in the first model when JO146 was added during persistence, it is tempting to suggest that penicillin persistence does not involve a detrimental level of extra-cytoplasmic protein stress. It is important to note that there is a possibility of some off-target impacts of JO146, however, given the marked phenotypes which correspond with very specific phases and conditions of chlamydial culture observed here, these impacts are likely minor. The absolute requirement for CtHtrA during recovery from penicillin persistence is an exciting finding, and to our knowledge is the first identification of an essential protein for this transition. In addition, JO146 treatment in the presence of heat stress in a time frame (4 h) was completely lethal. This is amazingly quick given 4 h is consistent with less than 2 rounds of binary fission for *C. trachomatis* serovar D [has been identified to take 2.4 h per round of binary fission (Miyairi et al., 2006)]. This suggests that CtHtrA is essential during certain stress conditions and does not necessarily relate to replication or binary fission. This data suggests that perhaps penicillin persistence is a strategy to reduce cellular and protein stress which may be an explanation for why CtHtrA was found not to be essential during penicillin persistence. Heat stress is highly likely to be a strong inducer of protein stress and for many bacteria is the main *in vitro* condition during which *htrA*- or *degP*- mutants are lethal (Lipinska et al., 1989). Therefore, it is not surprising that CtHtrA was essential even in this relatively short time of heat treatment. In summary, the data presented here demonstrates that the CtHtrA inhibitor JO146 is lethal for chlamydial recovery from penicillin persistence and for heat stress conditions.

## Author contributions

Vanissa A. Ong conducted the culture based experiments and analyzed data, James W. Marsh and Amba Lawrence conducted cultures experiments and analyzed data, Peter Timms contributed to experimental design and analyzed data. John A. Allan contributed to experimental design and analyzed data, Wilhelmina M. Huston contributed to experimental design and data analysis. All authors contributed to manuscript drafting.

### Conflict of interest statement

The authors declare that the research was conducted in the absence of any commercial or financial relationships that could be construed as a potential conflict of interest.
